# Inverse Model for the Control of Induction Heat Treatments

**DOI:** 10.3390/ma12172826

**Published:** 2019-09-02

**Authors:** Mohammad Zhian Asadzadeh, Peter Raninger, Petri Prevedel, Werner Ecker, Manfred Mücke

**Affiliations:** Materials Center Leoben Forschung GmbH (MCL), Roseggerstraße 12, A-8700 Leoben, Austria

**Keywords:** induction heating, heat transfer, model-based control, process control, inverse model, process modelling

## Abstract

In this work, we present and test an approach based on an inverse model applicable to the control of induction heat treatments. The inverse model is comprised of a simplified analytical forward model trained with experiments to predict and control the temperature of a location in a cylindrical sample starting from any initial temperature. We solve the coupled nonlinear electromagnetic-thermal problem, which contains a temperature dependent parameter α to correct the electromagnetic field on the surface of a cylinder, and as a result effectively the modeled temperature elsewhere in the sample. A calibrated model to the measurement data applied with the process information such as the operating power level, current, frequency, and temperature provides the basic ingredients to construct an inverse model toolbox, which finally enables us to conduct experiments with more specific goals. The input set values of the power supply, i.e., the power levels in the test rig control system, are determined within an iterative framework to reach specific target temperatures in prescribed times. We verify the concept on an induction heating test rig and provide two examples to illustrate the approach. The advantages of the method lie in its simplicity, computationally cost effectiveness and independence of a prior knowledge of the internal structure of power supplies.

## 1. Introduction

Induction heating is a complex multiphysics problem where electromagnetic, thermal, mechanical, and metallurgical problems are influencing each other. Therefore, modeling and simulation require the simultaneous consideration of complex interactions. The knowledge of the process variables and their complicated relationships is fundamental and necessary for simulation and modeling but not fully possible. Therefore, the process optimization and process control of induction heating and hardening is a true challenge both for the experimentalists and the theorists in the field.

There is a significant amount of literature and progressive study on the simulation and modeling of induction heating [1]. Numerical approaches such as the finite element (FE) and finite difference (FD) are quite popular for the analysis and modeling of coupled problems like induction heating. Since these approaches are based on discretization, the resulting differential equation system is very large, in the order of millions. Control approaches based on these methods, even if possible in principle, are challenging because they are computationally expensive and are not applicable when quick decision making is necessary. For example, the authors in Ref. [2] have studied and examined the efficiency of optimization approaches for induction heating processes based on the FE approach. They have focused on the power density distribution in the sample and the goal of achieving a prescribed temperature at a specific location near the surface of a cylindrical magnetic sample. Such models have the advantage of (1) a general description of physical mechanisms and (2) validity over a whole domain such as a sample or workpiece, as shown by [2] and other authors [3]. However, in face of the complexity of the algorithm presented in [2] and the usage of computationally expensive FE tools to solve electromagnetically-thermally coupled problems, the agreement between the optimized and targeted temperatures (Figure 12 of Ref. [2]) might not be sufficient for process optimization and especially process control concepts. Moreover, it is often necessary to consider the transient evolution of process parameters such as the current and frequency over the course of a heating cycle instead of assumed constant values. Model reduction is also an active research area for the control design and process control. In Ref. [4] the authors apply a reduced FE model for the control of the temperature profile of an axisymmetric sample by finding an optimal control input current. However, the comparison between the full and reduced model results shows an 80 K difference in the temperature at intermediate times. Therefore, applying a reduced model in real time and on induction heating test rigs to control the temperature profile will not perform satisfactorily.

In general, the mere application of physical approaches is not applicable to the induction heating systems because of the complexity of the power systems and the implemented control algorithms to the process control inputs. In real applications the induction heating operator is just allowed to change the power supply input control set values, while the control system, which is usually not accessible to the operator, will set the output physical parameters. To bypass this complexity and to propose a general approach applicable to any induction heating set up, and to avoid modeling of the power generator circuit, we present and apply an inverse model to control and optimize an inductive heat treatment process. The modeling strategy is not meant to replace a fully physical approach, but as a powerful alternative that is better suited for performance driven applications including interactions with the system control. With the implementation of such model types in the system control, the digital process control of induction heating may become a feasible task in the near future.

By combining a simplistic mathematical model with controlled experiments, we take into account the unknown relevant factors, (which are not explicitly included in the model, e.g., nonlinear boundary conditions, geometry imperfections, power generator complexities, etc.) that are important for the temperature evolution. In our induction heating test rig a cylindrical ferromagnetic sample is heated up above its Curie temperature in static experiments, where the inductor and sample are fixed. A mathematical model of the coupled electromagnetic-thermal problem is then calibrated with experiments to extract the temperature dependent model parameter α. The parameter α corrects the so-called modified Nagaoka coefficient which has mainly been assumed constant in the literature [5,6]. In principle, this parameter is temperature dependent and it changes during the heating process. However, its explicit functionality is not well known apart from some conjectures [7]. Therefore, we allow the optimization of this parameter by calibrating the model to the measurement data. In this way, the correct magnetic field in the air gap between the inductor and the sample is approximated and the modeled temperature is closer to the experiment. We also measure the current in the inductor and extract its frequency from the signal. Recording of the power level, current, frequency, temperature, and the model parameter α provides the basic ingredients to define an inverse model that enables us to predict and conduct experiments with specific goals. In industrial setups, due to instrumentation limitations, it is usually not feasible to continuously control the temperature of the samples applied to the induction heating processes. Moreover, the knowledge of the process control set values is necessary to target specific temperatures in specific times. The empirical way of trial and error is expensive, and the FEM approaches are computationally demanding or might even be impossible in industrial scales. To demonstrate the applicability of the presented approach for optimal control of induction heating processes we show two examples, where the target temperatures are chosen randomly to be achieved in specific times. Employing the inverse model we show how quickly and efficiently the target temperatures are reached by providing calculated control input set values to the power supply of our test rig.

The paper is organized as follows: In Section 2 we present the mathematical model and the solution of the coupled electromagnetic-thermal problem under certain assumptions. Section 3 shows the test rig setup for the experiments and in Section 4 the material properties of the heated sample are presented. In Section 5 we calibrate the forward model with experimental data and present some test simulations. In Section 6 we present an inverse model and as an application in Section 7 we demonstrate two experiments in which we desire to hit the prescribed target temperatures in specified times. Finally, in Section 8 we summarize and make a conclusion.

## 2. Mathematical Model

**Figure 1:**
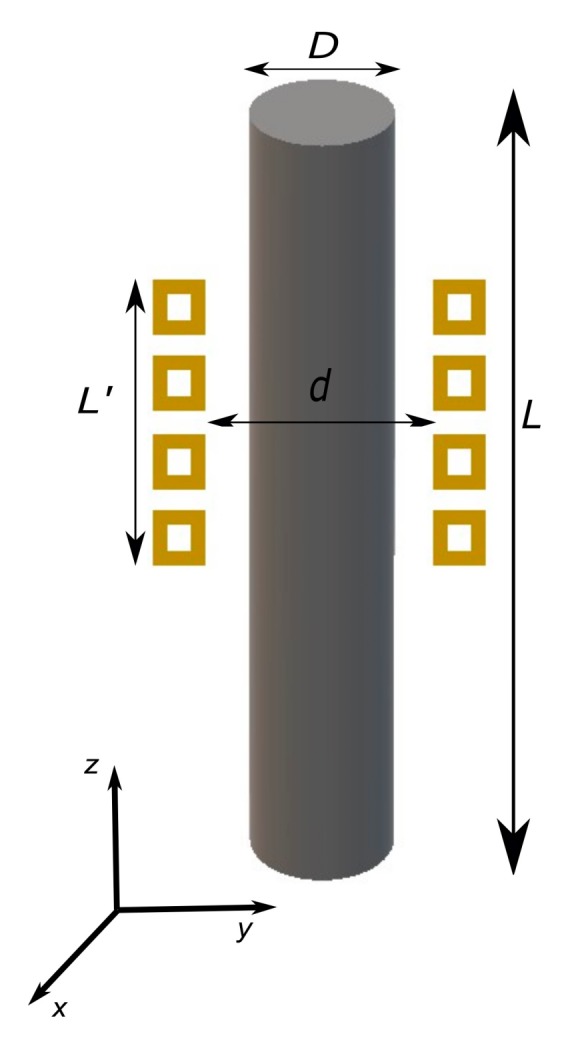
The geometries of the inductor and sample used in the mathematical model.

In Figure 1 the considered geometry used in the mathematical model is shown. The cylindrical conductor with length L and diameter D is heated up by a solenoid with an internal diameter d, length $L^{\prime }$ and rectangular cross section. In the following, we show the solution for the electromagnetic and thermal problem under specific assumptions.

### 2.1. Solution to the Electromagnetic Problem

Maxwell equations are the starting point to describe and model any kind of electromagnetic phenomena. These equations are as follows:

(1) $$\vec{\nabla }\times \vec{B}=0\;\vec{\nabla }\cdot \overset{\to }{\underset{-}{D}}=\rho_{c}\;\vec{\nabla }\times \vec{E}=-\frac{\partial \vec{B}}{\partial t}\;\;\vec{\nabla }\times \vec{H}=\vec{j}+\frac{\partial \overset{\to }{\underset{-}{D}}}{\partial t}\;$$

where $\vec{B}$ is the magnetic induction, $\vec{H}$ the magnetic field, $\vec{E}$ the electric field, $\overset{\to }{\underset{-}{D}}$ the electric flux density, $\vec{j}$ the electric current density and $\vec{\nabla }\times$ is the curl operator. The vector fields are also connected through material properties, for an isotropic material as

(2) $$\overset{\to }{\underset{-}{D}}=\epsilon \vec{E}\;\;\vec{B}=\mu \left(T,\left|\vec{H}\right|\right)\vec{H}\;\vec{j}=\sigma \left(T\right)\vec{E}$$

where ϵ is the dielectric constant, μ the magnetic permeability and σ the electrical conductivity. The set of Equations (1) and (2) is generic and applicable to any kind of geometry and system. Within certain assumptions, the analytical solution is possible. In this work we assume

(1) The inductor carries a sinusoidal current with frequency f and magnitude I.
(2) The magnetic field is purely axial, $\vec{H}=H\left(r\right)\hat{z}$, while the electric field is circumferential, $\vec{E}=E\left(r\right)\hat{\varphi }$.
(3) A homogenous medium with constant material properties.
(4) The displacement current is negligible, which is applicable to good conductors, $\frac{\partial \overset{\to }{\underset{-}{D}}}{\partial t}=0$, and frequencies below the megahertz range.
(5) Linear magnetic behaviour, $\vec{B}=\mu \vec{H}$
(6) No electric charge density, $\rho_{c}=0$

Assuming the aforementioned assumptions the magnetic field is described by the following differential equation [6]

(3) $$\frac{d^{2}H}{dr^{2}}+\frac{1}{r}\frac{dH}{dr}-i\omega \mu \sigma H=0\;$$

where ω=2πf is the angular frequency and $i=\sqrt{-1}$. The electric field can be calculated as follows

(4) $$E=-\frac{1}{\sigma }\frac{dH}{dr}$$

To find H from Equation (3) the value of the field on the surface of the cylinder ($H_{0}$) is required in the boundary conditions. For an ideal solenoid, the magnetic field is $H_{0}=NI/L\prime$ where N is the winding number. The value of the magnetic field for a short cylinder is corrected by the so-called modified Nagaoka coefficient $K_{n}$ [5,6]. The correction coefficient $K_{n}$ for a finite-length coil is expressed as

(5) $$K_{n}={\bar{K}}_{n}\left(1-\frac{D^{2}}{d^{2}}\right)+\frac{D^{2}}{d^{2}}\;{\bar{K}}_{n}=\frac{1+1.536\beta^{2}+0.274\beta^{4}}{1+1.036\beta^{2}}-\frac{8\beta }{3\pi }$$

where ${\bar{K}}_{n}$ is the Nagaoka coefficient and $\beta =\frac{d}{2L^{\prime }}$. The modified Nagaoka coefficient of Equation (5) just accounts for the geometry interaction corrections. This is a good approximation for a high frequency operating regime where the magnetic flux lines are mainly concentrated in the air gap between the solenoid and sample. However, in a low frequency regime, the magnetic flux substantially penetrates the sample and therefore the magnetic field becomes frequency dependent due to complex electromagnetic-thermal problem couplings. The authors in Ref. [7] introduce a frequency dependent (or temperature dependent) Nagaoka correction factor and verify its accuracy experimentally for different frequency ranges and sample materials. In this work we assume that the correction factor is a nonlinear function of temperature, which we determine numerically by calibrating the model with the experiments. The modified Nagaoka coefficient we consider in the model is

(6) $$K_{n}^{\prime }=\alpha \left(T\right)K_{n}$$

where $\alpha \left(T\right)$ is a temperature dependent parameter, which is found by calibrating the model with the experiments. This approach is more general and practical than the approach presented in Ref [7]. $\alpha \left(T\right)$ includes many other relevant influencing factors like geometry corrections (imperfections, complex shapes), material uncertainties, and boundary conditions. Therefore, the parameter  α(T) will cover the complex interactions of the relevant factors, which influence the field intensity in the air gap and as a result the Joule losses within the sample. We note that the temperature of the sample is a function of the control input variables (power level in our test rig) and material properties, which is not shown explicitly. This is due to the coupling of electromagnetic and thermal problems whose power dependency enters via the heat source term of the thermal equation (see Equations (10) and (11)). It is the objective of the presented approach to find the optimum process inputs (power levels) to achieve specific target temperatures (see details in Section 6).

The exact solution to Equation (3) is represented by the Bessel functions [6]

(7) $$\vec{H}=H_{0}\frac{J_{0}\left(\sqrt{-i}m\xi \right)}{J_{0}\left(\sqrt{-i}m\right)}\hat{z}$$

where $J_{0}\left(x\right)$ is the zero order Bessel function of the first type, $\xi =\frac{2r}{D}$, $m=\frac{\sqrt{2}D}{2\delta }$ and $H_{0}$ is the corrected field on the surface, i.e., $H_{0}=\frac{NIK_{n}^{\prime }}{L^{\prime }}$. The δ is the skin depth, i.e., the penetration depth of the magnetic field, which depends on the material properties and working frequency and is given by

(8) $$\delta =\sqrt{\frac{2}{\omega \mu \sigma }}$$

The active power flowing through the surface of the cylinder (W/m^2^) is given by the real part of the Poynting vector

(9) $$P=Re\left(\vec{E}\times \vec{H}\right)$$

The total amount of the heat per unit time that reaches the surface of the cylinder is

(10) $$q=\frac{P}{2}\pi DL$$

### 2.2. Solution to the Thermal Problem

The Fourier-Kirchhoff equation reads as

(11) $$\rho_{m}C_{p}\frac{\partial T}{\partial t}=\vec{\nabla }\cdot \left(\kappa \vec{\nabla }T\right)+q_{v}$$

where $\rho_{m}$ is the mass density, $C_{p}$ the specific heat at constant pressure, *κ* the thermal conductivity and $q_{v}$ is the energy rate per volume. In general, for the temperature distribution one has to solve the heat equation in the three-dimensional space. However, in this work for the sake of simplicity we assume that the sample is point like, such that the spatial gradients in the heat equation can be neglected. In principle, this assumption is valid for materials with high thermal conductivities. Furthermore, we assume that the cylinder heats up due to induction heating (Joule losses) and loses energy at the surface via radiation. We rewrite Equation (11) as

(12) $$\rho_{m}C_{p}\pi L\frac{D^{2}}{4}\frac{\partial T}{\partial t}=q-\pi \sigma_{B}\epsilon_{R}D\left(L+\frac{D}{2}\right)\left(T^{4}-T_{amb}^{4}\right)$$

where $\sigma_{B}$ is the Stefan–Boltzmann constant, $\epsilon_{R}$ the emissivity and $T_{amb}$ the ambient temperature. We notice that in Equation (12) q is the total heat generation rate, which is calculated from the electromagnetic solution and is given in Equation (10). This is the term that couples the thermal and electromagnetic problems. The temperature solution can be easily calculated over time using the explicit integration of Equation (12) with an initial temperature of $T\left(0\right)=T_{amb}$ (see details below).

### 2.3. Procedure to Solve Electromagneticlly-Thermally Coupled Problems

In this section, we show how in practice the electromagnetic-thermal problem is solved. We rewrite Equation (12) as

(13) $$\frac{\partial T}{\partial t}=aq\left(\alpha ,\delta \right)-b\left(T^{4}-T_{amb}^{4}\right)$$

where

(14) $$a=\frac{4}{\rho_{m}C_{P}\pi D^{2}L}\;b=\frac{4\sigma_{B}\epsilon_{R}}{\rho_{m}C_{P}D}\left(L+\frac{D}{2}\right)$$

The material properties $\left(C_{P,\;}\;\rho_{m}\right)$, the skin depth δ and the model parameter α are temperature dependent.

The total heat rate q depends on the yet unknown parameter α, which shall be determined by the calibration of the model to the experimental data. After using the explicit time integration, Equation (13) reads as

(15) $$T_{i+1}=T_{i}+dt\left(a_{i}q\left(\alpha_{i},\;\delta_{i}\right)-b_{i}\left(T_{i}^{4}-T_{amb}^{4}\right)\right)$$

where the subscript i refers to a point in time and dt is the integration time step. We also notice that $q\left(\alpha_{i},\;\delta_{i}\right)\equiv q\left(\alpha \left(T_{i}\right),\;\delta \left(T_{i}\right)\right)$. Since the temperature evolution is an initial value problem and the dynamic is causal, therefore the optimization is done at each time step. For each time step we have the cost function as

(16) $$cost=T_{i}+dt\left(a_{i}q\left(\alpha_{i},\;\delta_{i}\right)-b_{i}\left(T_{i}^{4}-T_{amb}^{4}\right)\right)-T_{experiment}$$

In this way, $\alpha_{i}$ is computed by fitting the model to the measurement at $t_{i+1}$. We use a standard built-in Scilab function for the optimization. Scilab is a free and open source software for numerical computations [8]. The “lsqrsolve” routine is used to minimize the sum of the squares of nonlinear functions (Levenberg–Marquardt algorithm).

**Figure 2:**
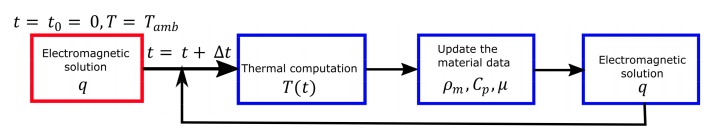
Flow chart of the procedure for solving coupled electromagnetic-thermal problems.

In Figure 2, we show the flow chart of how electromagnetic-thermal problems are solved once α is determined. First, we start (at $t=t_{0}$) by solving the electromagnetic problem at an ambient temperature to calculate the total heat generation, i.e., q. Then, in the next time step the thermal equation is integrated to get the new temperature. After the update of the material properties, we continue the loop by solving the electromagnetic problem and getting the new q. When we calibrate the model, the temperature computation is accompanied by an optimization to find α. We notice that for the analytical solution of the electromagnetic problem we assumed constant material properties, while in practice we allow for the temperature dependent material data. We believe this provides a very good approximation for the solution, and as mentioned previously, the temperature dependent Nagaoka coefficient comprises the missing factors as well.

## 3. Induction Heating Test Rig Setup

**Figure 3:**
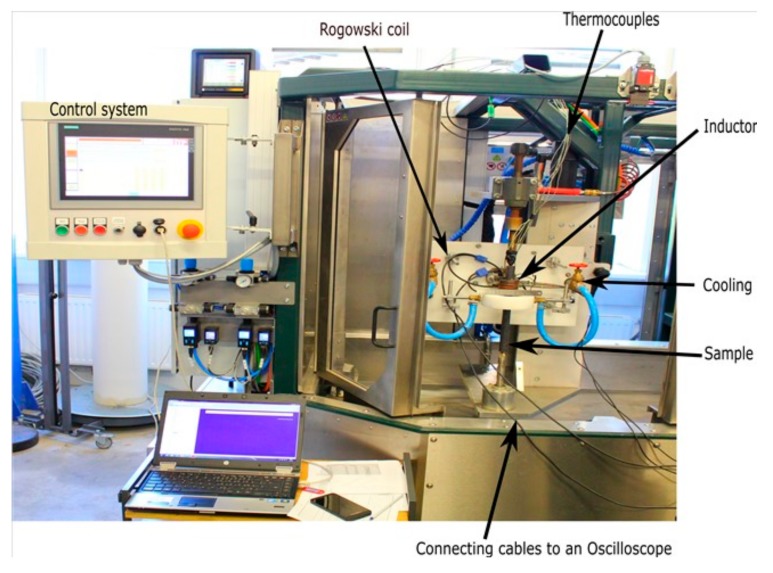
The induction heating test rig setup (product model: HU-VH300-MS30, manufacturer: Ideal Thermal Processes GmbH (ITP), city: Oberkirch, country: Germany).

The experimental setup is shown in Figure 3. The power supply operates both in the temperature control and power control mode with the capacity to provide a maximum power of 30 kW. The graphical user interface provides control over the operating power, heating time, movement of the inductor, rotation of the sample, and quenching. Both the water and air cooling are possible in the unit. Alternatively, the test rig can be controlled by means of the input files, which are loaded into the system control. In this way, heat treatment procedures with a high number of individual steps can be carried out.

The control system of the test rig provides the data of relevant process parameters during heating. These include the resonance frequency, running power, current, and voltage (inside the generator before the transformer, not in the inductor), temperature of the cooling water at the inlet and outlet of the solenoid, and the temperature of three points in the sample (two with thermocouples and one with a pyrometer or alternatively all three with thermocouples (NiCr-Ni, type K)). The recording of the data is performed with a sampling rate of 10 ms.

The cylinder of length L=300 mm and diameter D= 22 mm is fixed between two clamps. The rectangular inductor of length $L^{\prime }$ *=* 37 mm and internal diameter d=37 mm is used to heat up the part of the sample beneath the solenoid. The setup is static where neither the sample rotates nor the inductor moves. The point where the temperature is measured is located at a radial distance of r=2.1 mm from the surface and the thermocouple hole has a depth of 65 mm from the top side of the cylindrical sample. The position of the inductor is fixed such that the temperature measurement point (at the end of the thermocouple hole) is located at the center of the inductor. The diameter of the thermocouple measures 2.0 mm, while the hole has a diameter of 2.2 mm.

The current in the inductor is measured by implementing a Rogowski coil right after the transformer as shown in Figure 3. A Rogowski coil is wrapped around the straight conductor, which carries the current to the inductor. The induced voltage in a Rogowski coil is proportional to the rate of the current in the straight conductor. Therefore, the measured voltage is integrated to get the current as:

(17) $$I\left(t\right)=\frac{1}{M}\int_{t_{0}}^{t}V\left(t^{\prime }\right)dt^{\prime }$$

where M=0.064 µH is the mutual induction of the Rogowski coil and V is the voltage measured by an Oscilloscope attached to our test rig (to measure the voltage induced in the Rogowski coil). In principle, Rogowski coils are immune to external fields and show very low positioning related sensitivity. During our experiments this was checked and two different Rogowski coils have been used. The finally calculated currents from both Rogowski coils differed by a few amperes only. The frequency of the circuit is extracted from the time series of the current.

## 4. Material Properties

**Figure 4:**
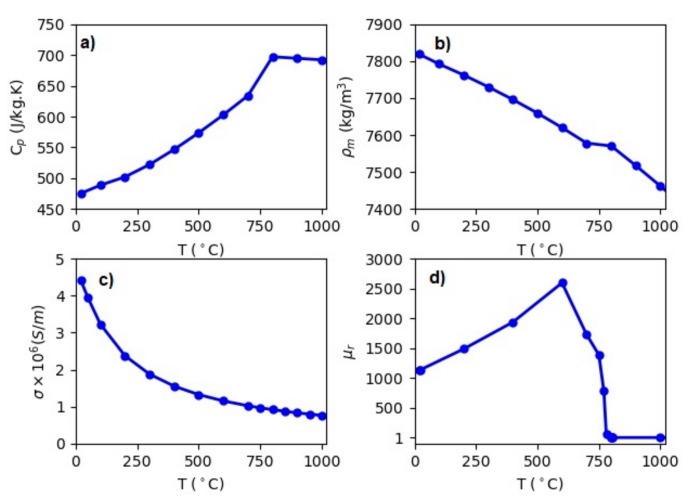
Material properties as a function of the temperature: (**a**) Specific heat, (**b**) mass density, (**c**) electrical conductivity, (**d**) relative magnetic permeability.

A sample material for the heating experiments of the steel grade 50CrMo4 was used with the chemical composition given in Table 1 [9]. In Figure 4, the specific heat, mass density, electrical conductivity, and relative magnetic permeability are shown compared to the temperature. These material data were modeled by with the “JMatPro” software (Version 9.0) [10]. Based on the calculated material data, the Curie temperature is around 800 °C.

## 5. Model Calibration and Testing

In this section, we check the accuracy of the mathematical model used for describing the experiments. To this end, we allow the optimization of only one parameter in the model. As described in Section 2, the optimized parameter corrects the magnetic field in the air gap between the cylindric sample and the inductor and even takes into account other effects that are not included in the model. For example, these are nonlinearity effects, inaccuracy of the material data, geometry asymmetries, and the effects originating from the test rig setup. 

We used a collection of experiments to calibrate the model. Table 2 shows the list of power variation experiments for the calculation of the model parameter α. The minimum and maximum powers in the measurements are 0.6 and 15 kW. We ran every experiment until the Curie temperature was exceeded. The repetitively used sample for the experiments was assured to be free of any cracks or other damages. To keep the sample in a good condition, we first conducted the experiments at low powers and water quenching was only applied after convective cooling of the sample in ambient air down to 400 °C. In this way, the sample does not experience extreme thermal and mechanical stresses. 

In the calibration stage we construct the tables of power, temperature, current, frequency, and the correction factor α. The only input control parameter in the experiments is the set value for the power in the control system. The current and frequency are determined by internal algorithms in the power supply, which depend on the sample temperature as well as the geometries of the sample and inductor. In the model, the current and frequency are taken from the Oscilloscope measurements. To find α in Equation (15), we use a fixed integration time step dt = 0.01 s that is equal to the sampling rate of the temperature measurement. The knowledge of $\alpha \left(T\right)$ for several powers and its corresponding currents and frequencies provides the basis to define an inverse model (see Section 6) with the ability to design experiments with specific goals, such as the predefined temperature evolution.

**Figure 5:**
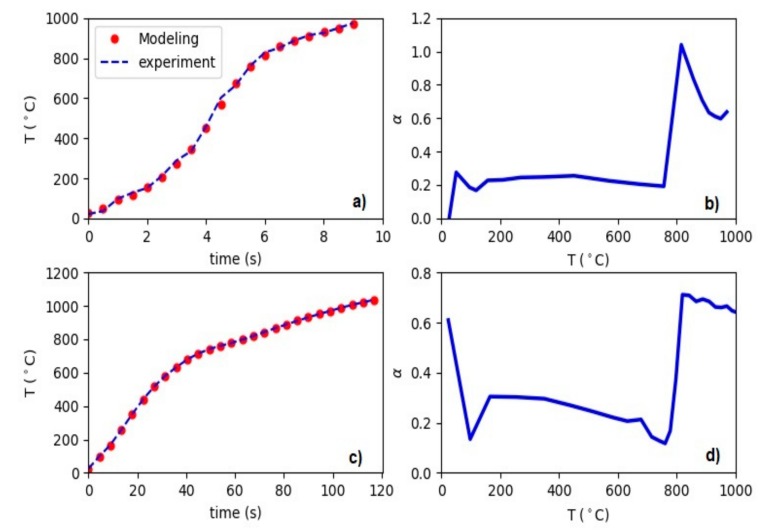
(**a**) Calibration of the model to the measurement data of the experiment done with 13.5 kW of power. (**b**) The optimized parameter α for the experiment with 13.5 kW of power. (**c**) Calibration of the model to the measurement data of the experiment done with 2.1 kW of power. (**d**) The optimized parameter α for the experiment with 2.1 kW of power.

Figure 5 illustrates the results of the model calibration for the power experiments with 13.5 and 2.1 kW and their corresponding optimized parameter α as a function of the temperature. We remind that the temperature is monitored at the measurement point of 2.1 mm below the surface. In principle, one could do this for any point in the sample. As we can see, with just one parameter the measurement data are accurately reproducible. With the increasing temperature, the evolution of α shows a transition zone corresponding to the Curie point of the material. For high powers, such as 13.5 kW, we see that α is almost constant while for low powers, such as 2.1 kW, it is decreasing while approaching the Curie temperature.

**Figure 6:**
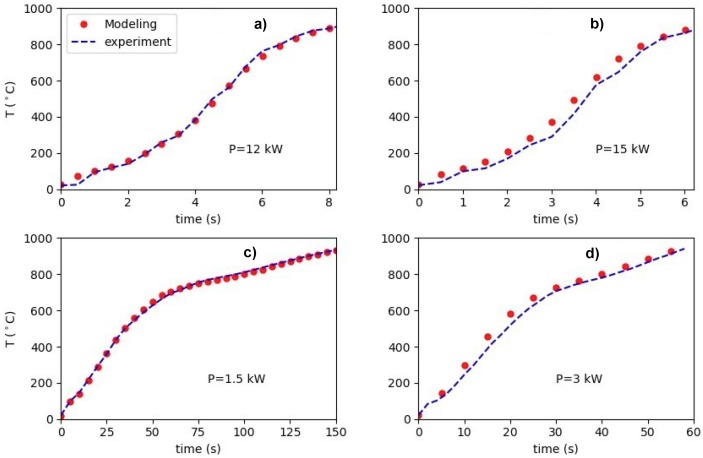
(**a**) Testing the model on the 12 kW measurements using the calibrated model from the 13.5 kW experiment. (**b**) Testing the model on the 15 kW measurements using the calibrated model from the 13.5 kW experiment. (**c**) Testing the model on the 1.5 kW measurement using the calibrated model from the 2.1 kW experiment. (**d**) Testing the model on the 3 kW measurement using the calibrated model from the 2.1 kW experiment.

After calibration, we test the model on some experiments. Figure 6 shows the accuracy of the calibrated model for four measurements different from the calibration set. In the upper panels of Figure 6 we show the results for 12 and 15 kW of the power experiments using the model calibrated with the 13.5 kW measurement data. As we can see, the simulated temperatures are very close to the experimental data. In the lower panels of Figure 6 the results are shown for 1.5 and 3 kW of the experiments using the model calibrated with the 2.1 kW measurement. Additionally, for the low power regime, the agreement is very promising. These examples show that using the α optimized for a specific power can provide a very accurate predictive model for experiments close to that power. This reflects the fact that *α* does not change substantially for the neighbouring power experiments. Therefore, in practice, for the model calibration a discrete set of experiments is required. Then, interpolation of the data will provide a database accurate enough for modeling.

## 6. Inverse Model

In this section, we present the inverse model and its application for control of the induction heating systems. The temperature control of the induction heating systems is a daunting task and up to now not completely managable. Direct temperature control is not possible in the industrial context since thermocouples can-not be applied in continuous processes and thermographic methods are not sufficiently reliable. A prior knowledge of the control set values to reach certain target temperatures in the sample in specific times has a great value for industrial setups and applications. Furthermore, optimal temperature control paves the way to hit desired targets in material properties as a final goal. The empirical way of the temperature adjustment is based on trial and error, which is not cost effective. Moreover, methods based on FEM simulations are computationally expensive and might be even impossible on industrial scales.

In our test rig power controlled static experiments, the only inputs to the control system are the power level and the total holding time. Therefore, the temperature of the sample at time *t* is a complicated function of power *P*, which can be expressed as

(18) $$T\left(t\right)=F\left(P\right)$$

The inverse problem is the process of finding power levels, which lead to certain temperatures in the sample, i.e.,

(19) $$P=F^{-1}\left(T\left(t\right)\right)$$

It is impossible to derive the explicit mathematical form of $F^{-1}$. However, employing a mathematical model, which is incorporated with some physical parameters, could assist to reverse engineer the problem. As described in Section 5, the unknown model parameters, α in this work, are determined by calibrating the model with the experiments. Performing the experiments with different power levels and recording the model parameters (current, frequency, α), the simulated temperature T and input set value P, provides a tool to bypass the necessity of knowing the explicit form of $F^{-1}$. With a discrete set of experiments and the interpolation of the data, we can infer the required input powers to reach specific temperatures. The linear interpolation (by using a standard Scilab built-in function [8]) has been done for three surfaces (α, current, frequency) of a matrix with the power level and temperature axes. The grid spacing of ΔP=0.03 kW and ΔT=0.1 °C is used in the interpolations.

To calculate the power levels with the model we start with an initial power level of $P_{n}$= 0.6 kW to simulate the temperature for a total time of $t_{n}-t_{n-1}$ by using Equation (15), where n is the index for numbering the target points (n = 1, 2, 3, ...). We notice that $(t_{0}=0,\;T\left(t_{0}\right)=T_{amb}$) and for multiple target temperatures the initial temperature in the simulations with Equation (15) for a target point n is $T\left(t_{n-1}\right)$.

The model parameters of the current, frequency and α are read from the interpolated surface data, based on the current values of the temperature and power level. The power level is changed in an iterative way to reach the target temperature, i.e.,

(20) $$P_{n}=P_{n}-0.01\times \left(T\left(t_{n}\right)-T_{n}\right)$$

where $T_{n}$ is the n-th target temperature and $T\left(t_{n}\right)$ is the simulated temperature after a total time $t_{n}-t_{n-1}$. The iteration is terminated when the target temperature is simulated within a tolerance of $\left|T\left(t_{n}\right)-T_{n}\right|<5$.

## 7. Inverse Model Application: Demonstration of the Temperature Control

**Figure 7:**
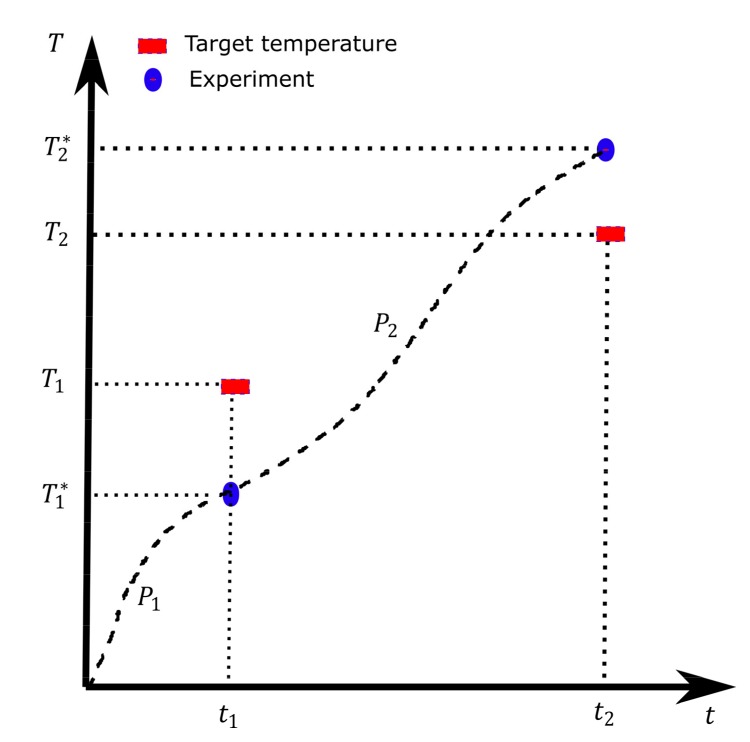
Schematics of one iteration (experiment) of the control assisted by the inverse model.

In principle, if the model is accurate enough, the initial powers calculated by the inverse model for reaching certain prescribed temperatures in the experiment might be good enough. However, in practice extra iteration loops with a few experiments might be required to reduce the target temperature error. In Figure 7 we show one iteration of this scenario where $T_{1}$ and $T_{2}$ are the target temperatures and $P_{1}$ and $P_{2}$ are the calculated initial powers by the inverse model as described in Section 6. When the model is imperfect, we reach other temperatures $T_{1}^{*}$ and $T_{2}^{*}$ (see Figure 7) in the experiment, which might be slightly higher ($T_{2}^{*}$ *>* $T_{2}$) or lower ($T_{1}^{*}$ *<* $T_{1}$*)* than the target temperatures. To get closer to the target temperatures, in the next experiment, a new set of input power levels is calculated by using the temperature values realized in the current experiment as target temperatures for the inverse model, i.e., $T_{1}^{*}$ and $T_{2}^{*}$. Then, we adjust the power levels for the next experiment depending on the target temperature error sign ($T_{i}-T_{i}^{*}$ *> 0* or < 0). We summarize the steps and the procedure for the update of the power levels as follows:

(1) Initial suggested powers ($P_{1}\;$ and $\;P_{2}$) found based on the inverse model to hit the target temperatures, i.e., $T_{1}$ and $T_{2}$ in Figure 7.
(2) Run the experiments with the suggested powers.
(3) Compare the temperatures ($T_{1}^{*}$ and $T_{2}^{*}$) with the target temperatures. Terminate the loop if the target is achieved within the specified accuracy, *|*$T_{i}-T_{i}^{*}$| < tolerance.
(4) If the target is not achieved, adjust the power levels. For the adjustment, the model calculates (in an iterative way as described in Section 6) the power levels to reach the new temperatures ($T_{1}^{*}$ and $T_{2}^{*}$) in step (3) and then suggests a set of power levels for the next iteration based on the difference between the new power levels and the old ones used in step (2), $P=P_{old}+\gamma \left|\Delta P\right|$. The γ is 0.03 or −0.03 depending on whether the measured temperature ($T_{i}^{*}$) is below or above the target temperature.
(5) Go to step (2).

**Figure 8:**
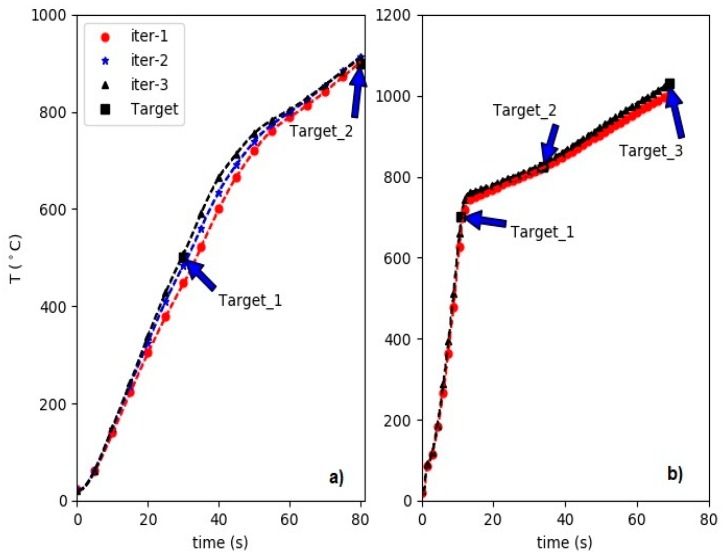
Illustration of two control experiments assisted by the inverse model. (**a**) Two target temperatures T = {500, 900} °C are set for times t = {30, 80} s. (**b**) Target temperatures T = {700, 825, 1030} °C are set for times t = {11, 34, 69} s.

**Figure 9:**
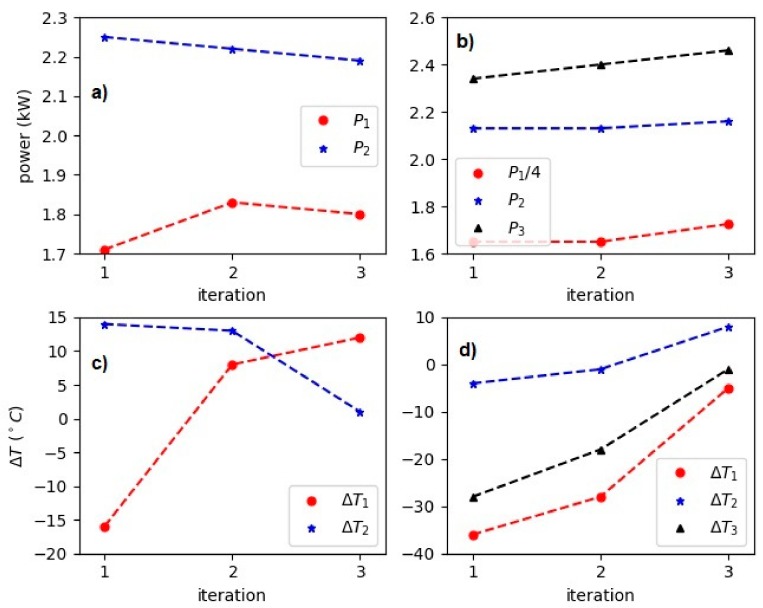
(**a**) Power level of each iteration for the control experiment with two target temperatures (T = {500, 900} °C). (**b**) Power level of each iteration for the control experiment with three target temperatures (T = {700, 825, 1030} °C). (**c**) Error in the target temperature for each iteration for the control experiment with two target temperatures. (**d**) Error in the target temperature for each iteration for the control experiment with three target temperatures.

As an application for the inverse model, we show the accuracy of the approach for designing targeted experiments in the induction heating systems. For the demonstration, we show two control experiments where we target two and three temperatures at specific times. In Figure 8 we show the results. Figure 8a shows the experiments to hit the temperatures T = {500, 900} °C at times t = {30, 80} s. In Figure 9 on the left panels the corresponding power levels (inferred by the inverse model) and errors in the target temperature are shown for each experiment. As we can see in three iterations the target temperatures are achieved within a tolerance of |$T_{i}-T_{i}^{*}$| < 12. Figure 8b shows the results for the second control experiment with iterations to hit the temperatures T = {700, 825, 1030} °C times t = {11, 34, 69} s. In addition, this control experiment converged in three iterations and we have omitted the iteration number two in the plot for better readability. As we can see in Figure 9 the power levels are not changing significantly during the iterations and already the first suggested powers lead to promising results. However, for better optimization and reducing the error (see Figure 9, lower panels) in the target temperatures a few iterations might be necessary. The control and design of experiments similar to the one in Figure 8b is very appealing to material scientists. In inductive heat treatments, the sample is heated up in a short time to achieve temperatures close to the Curie point and then the heating continues at a lower rate. A preliminary setup of such experiments and a good guess of the control input set values are crucial. The inverse model can substantially reduce time and energy for the design of such experiments.

Temperature control with smaller convergence tolerances is possible at the cost of increased experiment numbers. For higher accuracy and smaller tolerances each iteration should be done with a new sample. Since material properties are not changing significantly, the repeated use of a single sample is sufficient in order to show the principles of the presented method.

The optimization problem in this work may be a nonconvex one. In the application of the inverse model to find a control input power level, we always started the iterations with the lowest power, i.e., 0.6 kW. In this way, the iterative procedure most of the time converged to a global optimum. However, more advanced and general optimization approaches like the genetic algorithms [11] are preferable and might be necessary when the parameter search space is larger (more control input variables) or more complicated optimization constraints are imposed. 

## 8. Summary

The inverse model is used to conduct and control experiments which are very interesting for heat treatment applications. We performed two experiments with temperature targets at specific times during the course of heating. We demonstrated that by using the inverse model the target temperatures are hit within a few iterations. This is a significant improvement in comparison to the classic way of trial and error and even complicated mathematical optimization algorithms [2,12]. The method was only applied to a position close to the surface. However, it can easily be extended to multiple locations within the sample. 

Furthermore, the presented approach can be applied to a broad diversity of non-ideal complicated scenarios. For example, to the induction heating setups where the sample is either asymmetric (and has a complex geometry) or has non-ideal characteristics, or industrial configurations where an array of coils are combined to surface harden the samples. Most often the FEM approaches fail to simulate such large-scale processes due to the complexity of the boundary conditions and multiple uncontrolled process variables. 

In this work, the inverse model and the temperature control were demonstrated for static cases without the quenching or cooling steps. In principle, the inverse model can be extended to include quenching and cooling operations and even to take into account mechanical and metallurgical problems. Last but not least, the approach is computationally very efficient and that makes it an attractive alternative to control large scale induction heating systems.

The computational burden of our approach is in the order of min (1 to 10 min) and it grows quickly when the number of target temperatures is increased. The response surface models are quite useful for use in the system parameter identification and fast process control. These surrogate models [13] are applicable with a low computational effort to both linear and nonlinear problems. It is in the interest of authors to develop and investigate the neural network based metamodels (fast running surrogate models) to control and optimize the induction heat treatments.

## Figures and Tables

**Table 1:** The chemical composition of 50CrMo4 with values in the weight percent and Fe is balanced.

C	Mn	Cr	Mo	Si	P	S	Fe
0.49	0.71	1.05	0.18	0.27	0.016	0.01	bal

**Table 2:** List of power controlled static measurements for the calibration of the model.

$Power\left(P\right)$ **(kW)**	0.6	0.9	1.5	2.1	3.0	4.5	6.0	7.5	9.0	10.5	12.0	13.5	15.0
